# Association Between Frequency of Muscle-Strengthening Exercise and Depression Symptoms Among Middle and High School Students: Cross-Sectional Survey Study

**DOI:** 10.2196/50996

**Published:** 2024-04-17

**Authors:** Hao Wang, Huaidong Du, Yunqi Guan, Jieming Zhong, Na Li, Jin Pan, Min Yu

**Affiliations:** 1 Department of Noncommunicable Disease Control and Prevention Zhejiang Provincial Center for Disease Control and Prevention Hangzhou China; 2 Medical Research Council Population Health Research Unit Nuffield Department of Population Health University of Oxford Oxford United Kingdom; 3 Clinical Trial Service Unit & Epidemiological Studies Unit Nuffield Department of Population Health University of Oxford Oxford United Kingdom

**Keywords:** depression symptoms, muscle-strengthening exercise, adolescents, cross-sectional study

## Abstract

**Background:**

Existing literature on the association between the frequency of muscle-strengthening exercise (MSE) and depression among adolescents is limited and contradictory.

**Objective:**

This study aimed to elucidate the association of MSE frequency with depression symptoms among middle and high school students in China.

**Methods:**

A total of 27,070 students in grades 7-12 from 376 middle and high schools were surveyed using an anonymous self-administered questionnaire between April and June 2022. Information on engaging in MSE was self-reported, and depression symptoms were assessed using the Patient Health Questionnaire-9 (PHQ-9). Poisson regression was used to examine the association between MSE frequency and depression symptoms.

**Results:**

Among the 27,006 eligible students, 51.6% (n=13,933) were boys, and the mean age was 15.6 (SD 1.7) years. The overall prevalence of meeting MSE recommendations (ie, engaging in MSE ≥3 days/week) was 34.6% (95% CI 32.6%-36.6%; n=9145); the prevalence was higher in boys (43.8%, 95% CI 41.8%-45.8%; 6067/13,933) than in girls (24.3%, 95% CI 22%-26.6%; 3078/13,073; *P*<.001). A total of 5882 (21.8%) students reported having depression symptoms. After adjustment for sociodemographic status, lifestyle factors, academic performance, and experience of physical fighting, compared to students who did not engage in MSE, the prevalence ratios (PRs) for depression symptoms were 0.98 (95% CI 0.97-0.99) for those engaging in MSE once a week, 0.95 (95% CI 0.93-0.97) for 2 days/week, 0.93 (95% CI 0.90-0.96) for 3 days/week, 0.90 (95% CI 0.87-0.94) for 4 days/week, 0.88 (95% CI 0.84-0.93) for 5 days/week, 0.86 (95% CI 0.81-0.92) for 6 days/week, and 0.84 (95% CI 0.78-0.90) for 7 days/week, respectively.

**Conclusions:**

The overall prevalence of meeting MSE recommendations among Chinese adolescents is low. The frequency of MSE was inversely associated with depression symptoms.

## Introduction

Depression disorders account for a large and increasing health burden among adolescents aged 10 to 19 years worldwide [1]. The estimated number of disability-adjusted life years among adolescents aged 10 to 19 years diagnosed with depression disorders increased worldwide from 3.4 million to 4.3 million between 1990 and 2019 [2], and in 2019, there were 22.9 million adolescents aged 10 to 19 years with depression disorders worldwide, of whom 1.5 million were in China [2]. Depression among adolescents may contribute to many negative consequences, including functional impairment, poor cognitive development, poor academic performance, and suicide [3,4]. Depression is also associated with a wide variety of chronic physical disorders, including arthritis, asthma, cancer, cardiovascular disease, diabetes, obesity, hypertension, chronic respiratory disorders, and dementia, acting via multiple mechanisms that are not yet completely clear [5].

Muscle-strengthening exercise (MSE) is defined as physical exercise that increases skeletal muscle strength, power, endurance, and mass (eg, strength training, resistance training, or muscular strength and endurance exercises) [6]. Although MSE is now included in many national public health guidelines, in comparison to physical activity guidance on aerobic activities, there is still a lack of emphasis and guidance on MSE throughout public health policies in various countries [7-9]. Prevalence rates for meeting MSE recommendations (ie, engaging in MSE ≥3 days/week) among adolescents vary across countries and regions, as do secular trends. For instance, the prevalence rate of adhering to MSE guidelines is 44.9% in the United States [10], 53.7% in Canada [11], and 39.3% in China [12]. The prevalence in US adolescents decreased from 55.6% in 2011 to 49.5% in 2019 [13], while among Korean adolescents it slightly increased from 20.1% in 2009 to 21.9% in 2019 [14]. More recently, emerging epidemiological and clinical evidence has demonstrated that engagement in MSE could reduce the risk of chronic diseases, including obesity [14,15], hypertension [16], osteoporosis [17], and metabolic syndrome [18]; it can also enhance cardiometabolic health [19] and improve physical fitness [20].

Previous studies documented that MSE was inversely associated with depression. However, the majority of this research focused on adults [21-24], and little is known about adolescents. Furthermore, existing literature on the association of MSE frequency with depression among adolescents is contradictory. While one study indicated that meeting MSE recommendations was inversely associated with depression [25], a null association was found among US high school students and young adults aged 20-39 years [26,27]. Hence, this study was designed to evaluate the association between MSE frequency and depression symptoms among school students in China.

## Methods

### Study Design

This cross-sectional survey applied a multistage cluster sampling method to recruit participants. In stage 1, 30 counties or districts were sampled randomly from all 90 counties and districts in Zhejiang Province. In stage 2, 11 middle school classes, 6 academic high school classes, and 6 vocational high school classes were selected randomly within each chosen county or district. In stage 3, all students in the selected classes were invited to participate in the study. Students in the selected classes who also returned signed informed consent forms were included in the analysis unless they had serious health problems or illnesses that would restrict them from participating, including intellectual disabilities or language disorders.

A self-administered anonymous questionnaire was filled in by students in a classroom setting without school teachers’ supervision. The field survey was implemented by trained staff from county centers for disease control and prevention (CDC) using standardized procedures.

### Outcome Variables

The severity of depressive symptoms was assessed using the Patient Health Questionnaire-9 (PHQ-9) [28]. The PHQ-9, widely used among adolescents [29-32], is a brief scale designed to screen for symptoms of major depressive disorder within the past 2 weeks based on the codes of the *Diagnostic and Statistical Manual of Mental Disorders, Fifth Edition*. Participants were asked to score the following 9 items [33]: (1) “little interest or pleasure in doing things,” (2) “feeling down, depressed, or hopeless,” (3) “trouble falling or staying asleep, or sleeping too much,” (4) “feeling tired or having little energy,” (5) “poor appetite or overeating,” (6) “feeling bad about yourself,” (7) “trouble concentrating,” (8) “moving or speaking so slowly that other people could notice,” and (9) “thoughts that you would be better off dead or of hurting yourself.” Scores for each item ranged from 0 (not at all) to 3 (nearly every day); therefore, the total combined score ranged between 0 and 27. Following the current recommendation [28], depression was defined as having a total PHQ-9 score of no less than 10. A previous study documented high specificity (85%) and high sensitivity (88%) of the PHQ-9 scale in detecting depression using the cutoff value of 10 [34]. In addition, depression was divided into 4 groups according to the sum of the PHQ-9 score (5-9: mild depression; 10-14: moderate depression; 15-19: moderately severe depression; and 20-27: severe depression) [35].

### Exposure Variables

The frequency of engaging in MSE was evaluated through the question “During the past 7 days, on how many days did you do exercises to strengthen or tone your muscles, such as push-ups, sit-ups, or weight lifting?” Response options included “none,” “1 day,” “2 days,” “3 days,” “4 days,” “5 days,” “6 days,” and “7 days.” This item, with an acceptable reliability for children and adolescents (κ coefficient >0.55) [36], has been widely used for health behavior surveillance worldwide [10,11]. Meeting MSE recommendations was defined as engaging in MSE at least 3 days in the past 7 days, which is in accordance with World Health Organization (WHO) guidelines [6].

### Ethical Considerations

The study was approved by the Ethics Committee of the Zhejiang Provincial CDC (grant number 2022-007-01). The survey was anonymous, and participants did not need to write down their name on the questionnaire. Every student who finished the questionnaire received a gift as compensation (a pencil box). A written consent form was provided by all students and their guardians 2 weeks prior to the survey.

### Statistical Analysis

Continuous variables are presented as the mean (SD). Categorical variables are presented as the percentage (95% CI). Weighted prevalences were calculated. A modified Poisson regression, which is considered to be more robust than the traditionally used logistic regression [37], was used to examine the association between MSE frequency and depression symptoms [38]. Potential confounding factors comprised sociodemographic status, behavioral lifestyle factors, academic performance, and experience of physical fighting. Prevalence ratios (PRs) were calculated using two regression models: model 1 was adjusted for age (≤13 years, 14-15 years, or ≥16 years), sex (boy or girl), region (urban or rural), and type of school (middle school, academic high school, or vocational high school); model 2 was additionally adjusted for paternal and maternal education level (middle school or below, high school, or college or above), parental marital status (married or other), household income (very poor/poor, fair, or rich/very rich), cigarette smoking (yes or no), alcohol drinking (yes or no), physical activity (none, 1-2 days/week, 3-5 days/week, or 6-7 days/week), academic performance (excellent, middle, or poor), and being involved in physical fighting (yes or no). In addition, both exposure (ie, frequency of engaging in MSE) and outcome (ie, depression symptom score) variables were considered to be continuous variables, and multiple linear regression analyses were used to ascertain the association of days of engaging in MSE with depression symptom score. In sensitivity analyses, additional adjustment for experience of being bullied was performed. All statistical analyses were performed using SAS (version 9.4; SAS Institute). The statistical significance level was set at a *P* value <.05 using a 2-sided test.

## Results

### Descriptive Statistics

Overall, 28,043 students from 376 schools were invited and a total of 27,070 students participated in the survey (114 refused to participate and 859 were absent from school on the survey day), resulting in a response rate of 96.5%. Out of 27,070 students, a total of 64 were excluded because of either an incomplete questionnaire (n=40), missing at least 1 of the 9 items in the PHQ-9 questionnaire (n=17), or missing information on engaging in MSE (n=7). Ultimately, 27,006 students, consisting of 13,933 boys and 13,073 girls, were included in the current analyses (Figure S1 in Multimedia Appendix 1). The mean age was 15.6 (SD 1.7) years. A total of 21.8% (n=5882) of students reported experiencing depression symptoms. In addition, the prevalence of mild, moderate, moderately severe, and severe depression symptoms was 39.9% (95% CI 38.8%-40.9%; 10,902/27,006), 14.4% (95% CI 13.7%-15%; 3746/27,006), 5.3% (95% CI 5%-5.7%; 1407/27,006), and 2.7% (95% CI 2.4%-3%; 729/27,006), respectively (Table S1 in Multimedia Appendix 1).

Compared to students who did not perform MSE, students engaging in MSE were more likely to be young, male, from an urban area, attend middle school, live in an intact family, have parents educated to the college level or above, come from a high-income family, be physically active, achieve excellent academic performance, smoke cigarettes, and drink alcohol (Table 1).

**Table 1:** Participant characteristics by frequency of muscle-strengthening exercise (N=27,006). Percentages are weighted.

Characteristics	Frequency of muscle-strengthening exercise (days/week) among participants									*P* value for trend
	0 (n=10,703)	1 (n=3474)	2 (n=3684)	3 (n=2758)	4 (n=1300)	5 (n=1599)	6 (n=640)	7 (n=2848)		
Age (years), mean (SD)	16.1 (1.7)	15.6 (1.7)	15.5 (1.7)	15.3 (1.7)	15.4 (1.7)	15.2 (1.6)	15.5 (1.7)	15.2 (1.7)	<.001	
Girls, n (%)	6560 (60.1)	1802 (51.1)	1633 (43.4)	1131 (42.1)	440 (32.4)	580 (36)	209 (31.4)	718 (24.4)	<.001	
Rural, n (%)	6569 (66.3)	2072 (65.4)	2199 (65.5)	1613 (62.3)	794 (64.6)	891 (58)	395 (64.4)	1685 (62.8)	.002	
Middle school, n (%)	3701 (39.5)	1603 (50)	1840 (54.3)	1659 (64.2)	749 (60.9)	1045 (70)	361 (59.7)	1804 (67.1)	<.001	
Living in intact family, n (%)	9182 (86.3)	3047 (88.3)	3269 (89)	2432 (88.8)	1135 (87.4)	1371 (86.5)	572 (90.1)	2512 (88.6)	<.001	
Father with college education or above, n (%)	1687 (16.3)	624 (18.7)	626 (16.9)	563 (21.1)	249 (20.3)	322 (22.5)	150 (23.9)	672 (24.2)	<.001	
Mother with college education or above, n (%)	1571 (15.4)	597 (17.6)	618 (17.7)	542 (20.1)	229 (19)	319 (22.4)	147 (24.9)	626 (22.4)	<.001	
Family with high income, n (%)	725 (7)	266 (8.4)	304 (8.5)	244 (9.3)	115 (8.6)	170 (11.3)	81 (12.4)	385 (14.2)	<.001	
Physically active ≥6 d/wk, n (%)	791 (7.4)	306 (8.4)	510 (13.6)	552 (19.9)	286 (21.6)	388 (23.8)	295 (44.4)	1559 (54.2)	<.001	
Excellent academic performance, n (%)	1818 (16.8)	675 (19.3)	698 (18.9)	582 (20.5)	309 (24.8)	360 (23.1)	141 (22.7)	701 (25.1)	<.001	
Cigarette smoking, n (%)	370 (3.4)	135 (3.8)	166 (4)	130 (4.5)	53 (4.2)	79 (4.5)	22 (3)	159 (4.8)	<.001	
Alcohol drinking, n (%)	1668 (15.2)	569 (15.8)	612 (16.5)	462 (16.1)	220 (17.3)	277 (16.5)	109 (16.7)	544 (17.7)	<.001	
Physical fighting, n (%)	1078 (10.5)	477 (14.2)	529 (14.6)	409 (15.1)	205 (16.6)	250 (16.6)	98 (16.3)	530 (18.7)	<.001	

### Prevalence of Meeting MSE Recommendations

The prevalence of meeting MSE recommendations was 34.6% (95% CI 32.6%-36.6%; 9145/27,006) overall and 41.4% (95% CI 37.4%-45.5%), 42.2% (95% CI 38.3%-46.2%), and 25.2% (95% CI 23.3%-27.1%) for students aged ≤13 years, 14-15 years, and ≥16 years, respectively (*P*<.001). The prevalence was higher among boys (43.8%, 95% CI 41.8%-45.8%) than among girls (24.3%, 95% CI 22%-26.6%; *P*<.001). In addition, the prevalence among students attending middle school, academic high school, and vocational high school was 43.6% (95% CI 40.3%-46.9%), 25.5% (95% CI 23.2%-27.8%), and 24.1% (95% CI 21.3%-27%), respectively (*P*<.001) (Table 2).

**Table 2:** Weighted prevalence of meeting muscle-strengthening exercise recommendations by different characteristics.

Characteristics		Participants meeting recommendations/total participants (n/N)	Prevalence (95% CI)	*P* value	
**Age range (years)**					<.001
	≤13	2326/5594	41.4% (37.4%-45.5%)		
	14-15	3617/8575	42.2% (38.3%-46.2%)		
	≥16	3202/12,837	25.2% (23.3%-27.1%)		
**Gender**					<.001
	Boys	6067/13,933	43.8% (41.8%-45.8%)		
	Girls	3078/13,073	24.3% (22%-26.6%)		
**Area**					.14
	Urban	3767/10,788	37% (32.9%-41.1%)		
	Rural	5378/16,218	33.3% (30.8%-35.8%)		
**Type of school**					<.001
	Middle school	5618/12,762	43.6% (40.3%-46.9%)		
	Academic high school	1845/7373	25.5% (23.2%-27.8%)		
	Vocational high school	1682/6871	24.1% (21.3%-27%)		

### Association Between Frequency of Engaging in MSE and Depression Symptoms

After adjusting for sociodemographic factors, behavioral lifestyle, academic performance, and physical fighting, engaging in MSE was inversely associated with the prevalence of depression in an apparent exposure-response manner. Compared to those who did not take part in MSE, the PRs for depression symptoms were 0.98 (95% CI 0.97-0.99), 0.95 (95% CI 0.93-0.97), 0.93 (95% CI 0.90-0.96), 0.90 (95% CI 0.87-0.94), 0.88 (95% CI 0.84-0.93), 0.86 (95% CI 0.81-0.92), and 0.84 (95% CI 0.78-0.90), respectively, for those performing MSE for 1 to 7 days/week (Table 3). In multiple linear regression analyses, the adjusted β coefficient of the association between days of performing MSE and depression symptoms scores was –.05 (95% CI –.08 to –.02). An increment of 1 day of MSE was associated with a 0.05 decrease in PHQ-9 score among the adolescents (Table S2 in Multimedia Appendix 1).

**Table 3:** Adjusted prevalence ratios (PRs) of depression symptoms associated with frequency of muscle-strengthening exercise among students.

		Frequency of muscle-strengthening exercise, (days/week)									*P* value for trend
		0	1	2	3	4	5	6	7		
**Total**											
	Participants, n	10,703	3474	3684	2758	1300	1599	640	2848	N/A^a^	
	Depression, n	2724	743	717	506	227	298	123	544	N/A	
	Model 1^b^, PR (95% CI)	1 (reference)	0.97 (0.96-0.98)	0.95 (0.92-0.97)	0.92 (0.89-0.95)	0.89 (0.85-0.93)	0.87 (0.82-0.92)	0.84 (0.79-0.90)	0.82 (0.76-0.89)	<.001	
	Model 2^c^, PR (95% CI)	1 (reference)	0.98 (0.97-0.99)	0.95 (0.93-0.97)	0.93 (0.90-0.96)	0.90 (0.87-0.94)	0.88 (0.84-0.93)	0.86 (0.81-0.92)	0.84 (0.78-0.90)	<.001	
**Boys**											
	Participants, n	4143	1672	2051	1627	860	1019	431	2130	N/A	
	Depression, n	862	285	329	236	118	162	77	337	N/A	
	Model 1, PR (95% CI)	1 (reference)	0.98 (0.97-0.99)	0.96 (0.93-0.99)	0.94 (0.90-0.98)	0.92 (0.87-0.97)	0.90 (0.84-0.97)	0.88 (0.81-0.96)	0.87 (0.78-0.96)	<.001	
	Model 2, PR (95% CI)	1 (reference)	0.97 (0.95-0.98)	0.94 (0.91-0.96)	0.91 (0.87-0.95)	0.88 (0.82-0.93)	0.85 (0.78-0.91)	0.82 (0.75-0.90)	0.79 (0.71-0.88)	<.001	
**Girls**											
	Participants, n	6560	1802	1633	1131	440	580	209	718	N/A	
	Depression, n	1862	458	388	270	109	136	46	207	N/A	
	Model 1, PR (95% CI)	1 (reference)	0.98 (0.96-0.99)	0.95 (0.93-0.99)	0.93 (0.89-0.98)	0.91 (0.86-0.97)	0.89 (0.82-0.96)	0.87 (0.79-0.96)	0.85 (0.76-0.95)	<.001	
	Model 2, PR (95% CI)	1 (reference)	0.98 (0.97-0.99)	0.96 (0.94-0.99)	0.95 (0.91-0.99)	0.93 (0.88-0.99)	0.91 (0.85-0.98)	0.90 (0.82-0.98)	0.88 (0.80-0.98)	<.001	

^a^N/A: not applicable.

^b^Model 1: adjusted for age, gender, region, and type of school.

^c^Model 2: adjusted for age, gender, region, type of school, parental education level, parental marital status, family income, cigarette smoking, alcohol drinking, physical activity, academic performance, and physical fighting.

### Sensitivity Analyses

The sensitivity analysis (ie, additional adjustment for experience of being bullied) did not essentially change the association of engaging in MSE with depression symptoms (Table S3 in Multimedia Appendix 1).

## Discussion

### Principal Findings

To our knowledge, this is the ﬁrst study examining the association between MSE frequency and depression in a provincially representative sample of Chinese middle and high school students. Our findings show that performing MSE was associated with a lower prevalence of depression symptoms among adolescents. In addition, our data also provide the latest information on the prevalence of meeting the MSE recommendations among Chinese adolescents.

### Prevalence of Meeting MSE Recommendations

In this study, the overall prevalence of meeting MSE recommendations was 34.6%, much lower than in other studies performed in high-income countries and a previous study conducted in China. For instance, one nationally representative survey conducted in 2021 among 17,232 US students reported that 44.9% of high school students exercised ≥3 days/week to strengthen or tone their muscles [10]. Another study conducted in 2013 and 2014 of 35,297 grade 9-12 Canadian students reported that 53.7% of students performed MSE ≥3 days per week [11]. One nationally representative survey implemented in 2019 of 56,636 Chinese middle and high school students found that 43.4% of middle school students and 28.1% of high school students met the WHO MSE recommendations [12]. Physical activity guidelines for Chinese people published in 2021 recommend that children and adolescents aged 6-17 years should engage in moderate- to vigorous-intensity physical activity at least 60 minutes daily and engage in MSE at least 3 days a week [39]. The Chinese government has formulated many regulations to ensure that there are at least 3 physical education (PE) classes per week for middle school students and at least 2 PE classes per week for high school students [40]. However, each 45-minute PE class in China comprises learning and training for basic sports skills, confrontational competitions, and muscle stretching and relaxing [40], and MSE was not specifically emphasized for PE classes. Hence, a closer focus should be placed on MSE, and consideration should be given to incorporating it into PE classes as an important component in the future.

Correlates of meeting the MSE recommendations observed in this study included household income, parental education level, and overall physical activity level. This is similar to findings from a report from Xin et al [12] showing that students who were from low-income households, had poorly-educated parents, and were physically inactive were less likely to meet the MSE recommendations. Consistent with this previous study [12], the prevalence of meeting MSE recommendations was lower among high school students than among middle school students. Also in line with previous studies [12,13], the prevalence of meeting MSE recommendations was lower among girls than among boys. In contrast to the study by Miller et al [41], which found a null significant association between cigarette smoking and meeting the MSE recommendations among 8383 adolescents aged 12-17 years, the more frequently students engaged in MSE, the more likely they were to smoke and drink in this study.

### Association Between Frequency of Engaging in MSE and Depression Symptoms

Although an accumulating body of research has focused on the association of engaging in MSE with depression symptoms, the results remain conflicting and inconclusive. In this study, MSE frequency was inversely associated with depression symptoms among adolescents, which is consistent with previous findings. For example, in a cross-sectional study of 411,080 US adults aged ≥18 years without any moderate-to-vigorous intensity physical activity, engaging in MSE was significantly and inversely associated with the prevalence of clinically diagnosed depression, with a PR of 0.89 (95% CI 0.85-0.93) for MSE 3 times/week and 0.89 (95% CI 0.85-0.93) for MSE ≥4 times/week in comparison to those did not engage in MSE [21]. Another cross-sectional study of 601 Irish participants aged ≥18 years observed that higher frequency and intensity of MSE protected against depression [42]. However, results from a nationally representative cross-sectional study of 13,677 US high school students in grades 9-12 indicated no statistical signiﬁcance in the association of engaging in MSE ≥3 days/week with depression symptoms [26]. Another study of 2088 US participants aged 20-39 years, with data retrieved from the National Health and Nutrition Examination Survey, demonstrated that it was physical activity, not MSE, that was independently associated with depressive symptoms [27].

Interestingly, several cohort studies have documented that handgrip strength was inversely associated with the incidence of depression [43-45]. For example, a UK Biobank prospective cohort study of 162,167 participants with a median follow-up of 10 years indicated that compared with participants in the highest tertile of handgrip strength, those in the medium and lowest tertiles had an 11% (hazard ratio [HR] 1.11, 95% CI 1.04-1.19) and 24% (HR 1.24, 95% CI 1.16-1.33) higher risk of depression, respectively [43]. Handgrip strength is often recognized as an indicator of overall muscle strength [46].

The underpinning mechanisms through which engaging in MSE could reduce the development of depression symptoms are largely unclear. First, engaging in MSE could improve muscle strength, which has been recognized as a health indicator and constituent of physical fitness, and enhance physical function and quality of life [20,47]. Second, exercise could increase feelings of self-efficacy and self-esteem, which may contribute to preventing mental health disorders [47,48].

The findings of this study are of practical public health importance and provide vital evidence that may inform the assessment of MSE and prevention of adolescent depression. First, only one-third of middle and high school students met the MSE recommendations, suggesting that insufficient MSE prevails among students of this age, and more comprehensive and effective efforts are needed to address these issues in China. These might include raising social awareness of the beneficial effect of engaging in MSE, incorporating MSE into PE classes, encouraging students to engage in MSE regularly, and evaluating the effects of MSE on physical and mental health among adolescents. Second, the American Heart Association updated a statement in September 2023 to clearly state that MSE is a safe, effective, and essential component of the overall physical activity regimen for cardiovascular disease risk reduction [49]. This study fills a gap in knowledge of the association of MSE frequency and depression symptoms among Chinese adolescents.

### Strengths and Limitations

The strengths of this study included a large sample size, a high response rate, and a standardized procedure. The study also had several limitations. First, because the study was cross-sectional in design, engaging in MSE and depression symptoms cannot be temporally ordered. It is highly possible that bidirectional associations exist between MSE and depression, because adolescents who have depressive symptoms may be less likely to engage in MSE. Large prospective studies using genetic tools (eg, Mendelian randomization methods) may help with clarifying the association structure between these 2 factors. Second, we only collected the number of days of engaging in MSE in the past week and did not collect detailed information on intensity and duration of MSE. Third, all data were self-reported, and the findings presented may be susceptible to recall or social desirability biases.

### Conclusions

In summary, our study sheds light on the association between MSE frequency and depression symptoms among middle and high school students in China. We found that the prevalence of meeting MSE recommendations was low, and that frequency of MSE was inversely associated with depression symptoms among students, although the exact direction of the association could not be determined. Once approved, it would be appropriate to incorporate MSE into methods for targeted prevention of adolescent depression.

## Appendix

Multimedia Appendix 1 Supplemental figures and tables.

## Abbreviations

CDC: county center for disease control and prevention
HR: hazard ratio
MSE: muscle-strengthening exercise
PE: physical education
PHQ-9: Patient Health Questionnaire-9
PR: prevalence ratio
WHO: World Health Organization

## Data Availability

The data sets used and/or analyzed during this study are available from the corresponding author on reasonable request.
