# Supplementary material for: Association Between Frequency of Muscle-Strengthening Exercise and Depression Symptoms Among Middle and High School Students: Cross-Sectional Survey Study
Source: JMIR Public Health Surveill. 2024 Apr 17;10:e50996. doi: 10.2196/50996 (PMC11063876; doi:10.2196/50996)
Supplement: Multimedia Appendix 1 [file publichealth_v10i1e50996_app1.docx]

**Multimedia Appendix 1**

**Contents**

**Figure S1. Study flow diagram**........................................................................................2

**Table S1 Weighted prevalence of mild, moderate, moderately severe, and severe depression symptoms by different characteristics**......................................................3

**Table S2 Adjusted β coefficients for scores of depression symptoms associated with muscle-strengthening exercise among students**.................................................4

**Table S3 Association of depression symptoms with muscle-strengthening exercise after further adjustment for being bullied**.....................................................................5

28,043 students from 376 schools were invited

27,070 students were surveyed

27,006 students were included in the final analyses.

114 refused to participate

859 absent from school on the survey

64 were excluded:

Incomplete questionnaire (n=40)

Missing information on PHQ-9 (n=17)

Missing information on MSE (n=7)

**Figure S1. Study flow diagram**

**Table S1 Weighted prevalence of mild, moderate, moderately severe, and severe depression symptoms by different characteristics**

|  | Participants, n | Mild | | |  | Moderate | | |  | Moderately severe | | |  | Severe | | |
| --- | --- | --- | --- | --- | --- | --- | --- | --- | --- | --- | --- | --- | --- | --- | --- | --- |
|  |  | Case, n | Prevalence  (95%CI) | *P* value |  | Case, n | Prevalence  (95%CI) | *P* value |  | Case, n | Prevalence  (95%CI) | *P* value |  | Case, n | Prevalence  (95%CI) | *P* value |
| Age range (years) |  |  |  | <.001 |  |  |  | .01 |  |  |  | .56 |  |  |  | <.001 |
| ≤13 | 5594 | 1825 | 32.4  (30.4-34.4) |  |  | 675 | 12.7  (11.4-14.0) |  |  | 265 | 5.1  (4.5-5.7) |  |  | 137 | 2.4  (1.9-2.8) |  |
| 14-15 | 8575 | 3264 | 37.5  (36.0-39.1) |  |  | 1219 | 14.9  (13.9-15.9) |  |  | 464 | 5.2  (4.6-5.9) |  |  | 269 | 3.4  (2.9-4.0) |  |
| ≥16 | 12,837 | 5813 | 45.5  (44.2-46.7) |  |  | 1852 | 14.8  (13.8-15.8) |  |  | 678 | 5.5  (5.0-6.0) |  |  | 323 | 2.4  (2.0-2.8) |  |
| Gender |  |  |  | <.001 |  |  |  | <.001 |  |  |  | <.001 |  |  |  | <.001 |
| Boys | 13,933 | 5312 | 37.8  (36.5-39.1) |  |  | 1608 | 12.0  (11.3-12.7) |  |  | 543 | 3.9  (3.5-4.3) |  |  | 255 | 1.9  (1.5-2.2) |  |
| Girls | 13,073 | 5590 | 42.2  (40.9-43.5) |  |  | 2138 | 17.0  (16.1-18.0) |  |  | 864 | 6.9  (6.3-7.4) |  |  | 474 | 3.7  (3.3-4.1) |  |
| Area |  |  |  | 0.61 |  |  |  | .003 |  |  |  | <.001 |  |  |  | .006 |
| Urban | 10,788 | 4324 | 39.4  (37.6-41.3) |  |  | 1421 | 13.2  (12.4-14.0) |  |  | 482 | 4.4  (3.9-4.8) |  |  | 249 | 2.3  (2.0-2.6) |  |
| Rural | 16,218 | 6578 | 40.1  (38.5-41.7) |  |  | 2325 | 15.0  (14.1-15.9) |  |  | 925 | 5.9  (5.4-6.3) |  |  | 480 | 3.0  (2.6-3.4) |  |
| Type of school |  |  |  | <.001 |  |  |  | .02 |  |  |  | .10 |  |  |  | .09 |
| Middle school | 12,762 | 4474 | 34.9  (33.4-36.4) |  |  | 1692 | 13.9  (13.0-14.8) |  |  | 642 | 5.1  (4.6-5.6) |  |  | 362 | 3.0  (2.6-3.4) |  |
| Academic high school | 7373 | 3555 | 48.0  (46.2-49.7) |  |  | 1146 | 15.9  (14.5-17.4) |  |  | 444 | 6.0  (5.3-6.7) |  |  | 211 | 2.6  (2.1-3.1) |  |
| Vocational high school | 6871 | 2873 | 42.0  (40.4-43.6) |  |  | 908 | 13.6  (12.2-14.9) |  |  | 321 | 5.0  (4.1-5.8) |  |  | 156 | 2.3  (1.7-2.8) |  |

**Table S2 Adjusted β coefficients for scores of depression symptoms associated with muscle-strengthening exercise among students**

|  | Adjusted β^a^ (95%CI) | *P* value |
| --- | --- | --- |
| Total |  |  |
| Model 1^b^ | -0.10 (-0.12, -0.07) | <.001 |
| Model 2^c^ | -0.05 (-0.08, -0.02) | <.001 |
| Boys |  |  |
| Model 1^b^ | -0.10 (-0.13, -0.07) | <.001 |
| Model 2^c^ | -0.05 (-0.08, -0.02) | .002 |
| Girls |  |  |
| Model 1^b^ | -0.10 (-0.15, -0.06) | <.001 |
| Model 2^c^ | -0.05 (-0.10, -0.01) | 0.02 |

^a^β coefficients (95%CI) represent changes in the scores of depression symptoms per day difference in muscle-strengthening exercise.

^b^Model 1: adjusted for age, gender, region, and type of school.

^c^Model 2: adjusted for age, gender, region, type of school, parental education level, parental marital status, family income, cigarette smoking, alcohol drinking, physical activity, academic performance, and physical fight.

**Table S3 Association of depression symptoms with frequency of muscle-strengthening exercise after further adjustment for being bullied**

|  | Frequency of muscle-strengthening exercise | | | | | | | | *P* for trend |
| --- | --- | --- | --- | --- | --- | --- | --- | --- | --- |
|  | None | 1 day/wk^d^ | 2 days/wk | 3 days/wk | 4 days/wk | 5 days/wk | 6 days/wk | 7 days/wk |  |
| Total |  |  |  |  |  |  |  |  |  |
| Participants, n | 10,703 | 3474 | 3684 | 2758 | 1300 | 1599 | 640 | 2848 |  |
| Depression, n | 2724 | 743 | 717 | 506 | 227 | 298 | 123 | 544 |  |
| APR^a^ (95%CI^b^) | 1(ref^c^) | 0.98  (0.97-0.99) | 0.96  (0.94-0.97) | 0.93  (0.91-0.96) | 0.91  (0.88-0.95) | 0.89  (0.85-0.93) | 0.87  (0.83-0.92) | 0.85  (0.80-0.91) | <.001 |
| Boys |  |  |  |  |  |  |  |  |  |
| Participants, n | 4143 | 1672 | 2051 | 1627 | 860 | 1019 | 431 | 2130 |  |
| Depression, n | 862 | 285 | 329 | 236 | 118 | 162 | 77 | 337 |  |
| APR^a^ (95%CI) | 1(ref) | 0.97  (0.96-0.98) | 0.94  (0.92-0.97) | 0.92  (0.88-0.95) | 0.89  (0.85-0.94) | 0.87  (0.81-0.92) | 0.84  (0.78-0.91) | 0.82  (0.75-0.90) | <.001 |
| Girls |  |  |  |  |  |  |  |  |  |
| Participants, n | 6560 | 1802 | 1633 | 1131 | 440 | 580 | 209 | 718 |  |
| Depression, n | 1862 | 458 | 388 | 270 | 109 | 136 | 46 | 207 |  |
| APR^a^ (95%CI) | 1(ref) | 0.98  (0.97-0.99) | 0.96  (0.94-0.99) | 0.95  (0.91-0.99) | 0.93  (0.89-0.98) | 0.91  (0.86-0.97) | 0.90  (0.83-0.97) | 0.88  (0.81-0.96) | <.001 |

^a^APR: Adjusted prevalence ratio. Prevalence ratios were adjusted for age, gender, region, type of school, parental education level, parental marital status, family income, cigarette smoking, alcohol drinking, physical activity, academic performance, physical fight, and being bullied.

^b^CI: confidence interval.

^c^ref: reference.

^d^wk: week.
